# Assessing Medical Student Readiness to Navigate Language Barriers in Telehealth: Cross-sectional Survey Study

**DOI:** 10.2196/36096

**Published:** 2022-08-11

**Authors:** Leena Yin, Fiona Ng, Mateo Rutherford-Rojas, Mia Williams, Susannah Cornes, Alicia Fernandez, Maria E Garcia, Elaine C Khoong

**Affiliations:** 1 School of Medicine University of California, San Francisco San Francisco, CA United States; 2 Department of Family Medicine Swedish Cherry Hill Hospital Seattle, WA United States; 3 Department of Medicine University of California, San Francisco San Francisco, CA United States; 4 Interpreting, Translation and Language Services UCSF Health San Francisco, CA United States; 5 Division of General Internal Medicine Department of Medicine University of California, San Francisco San Francisco, CA United States; 6 Weill Institute for Neurosciences University of California, San Francisco San Francisco, CA United States; 7 Division of General Internal Medicine at Zuckerberg San Francisco General Hospital Department of Medicine University of California, San Francisco San Francisco, CA United States; 8 UCSF Center for Vulnerable Populations at Zuckerberg San Francisco General Hospital San Francisco, CA United States; 9 Multiethnic Health Equity Research Center University of California, San Francisco San Francisco, CA United States

**Keywords:** interpreters, language barriers, medical students, medical education, limited English proficiency, telehealth, telemedicine, online education, clinician, health care professional

## Abstract

**Background:**

The COVID-19 pandemic has greatly increased telehealth usage in the United States. Patients with limited English proficiency (LEP) face barriers to health care, which may be mitigated when providers work with professional interpreters. However, telehealth may exacerbate disparities if clinicians are not trained to work with interpreters in that setting. Although medical students are now involved in telehealth on an unprecedented scale, no educational innovations have been published that focus on digital care across language barriers.

**Objective:**

The aim of this study is to investigate advanced medical students’ confidence in caring for patients with LEP during telehealth encounters.

**Methods:**

We administered a written survey to medical students on clinical clerkships at one US institution in August and September 2020. We assessed students’ overall confidence in working with interpreters; confidence in performing 8 clinical tasks during in-person versus telehealth encounters; and frequency of performing 5 different clinical tasks with patients with LEP compared to English-speaking patients during in-person versus telehealth encounters. Wilcoxon signed-rank tests and chi-square tests were used to compare confidence and task performance frequency, respectively, for patients with LEP versus English-speaking patients during telehealth encounters. Students were also asked to identify barriers to care for patients with LEP. The free-response questions were qualitatively analyzed using open coding to identify key themes.

**Results:**

Of 300 medical students surveyed, 121 responded. Furthermore, 72 students answered >50% of questions and were included in the analyses. Compared to caring for patients with LEP during in-person encounters, respondents were less confident in working with interpreters (*P*<.001), developing trust (*P*<.001), identifying agenda (*P*=.005), eliciting preferences for diabetes management (*P*=.01), and empowering patients in lifestyle modifications (*P*=.04) during telehealth encounters. During both in-person and telehealth encounters, approximately half of students (40%-78%) reported engaging less frequently in every clinical task with patients with LEP and this was as low as 22% (13/59) for some tasks. Students identified these key barriers to care for patients with LEP: time pressure, interpretation quality and access, technical difficulties, cultural differences, and difficulty with rapport building.

**Conclusions:**

Advanced medical students were significantly less confident caring for patients with LEP via telehealth than in person. Broader implementation of training around navigating language barriers is necessary for telehealth care, which has rapidly expanded in the United States. Our study identified potential key areas for curricular focus, including creating patient-centered agendas and management plans within the constraints of virtual settings. These developments must take place simultaneously with systems-level improvements in interpreter infrastructure to ensure high-quality care for linguistically diverse patients.

## Introduction

In the last two years, we have seen a massive increase in telehealth use as hospitals and clinics work to minimize COVID-19 transmission [1]. In the United States alone, one study estimated that usage has increased by 8336% from prepandemic levels [2]. These changes, which will likely last beyond the pandemic [3], have the potential to broaden access to care and decrease health care costs; however, they may also widen existing disparities [4-6].

Decades of research have shown that patients with limited English proficiency (LEP), who comprise 8% of the US population [7], have poorer health outcomes compared with their English-speaking counterparts. These outcomes, ranging from hospital admission to medication-related adverse events [8], can be partially explained by worse access to care [9-11]. For telehealth, patients with LEP had lower rates of use than proficient English speakers even before the pandemic [12]. This gap has persisted throughout the pandemic-driven telehealth expansion [13,14]. Additionally, patients with LEP who do access care, even in traditional modalities, may continue to experience poorer outcomes unless seen by a language-concordant provider or a provider working with a professional interpreter [15-17], which is not always the case. In one national study, 40% of ambulatory physicians reported never working with professional interpreters for their patients with LEP [18].

Formal training around care for patients with LEP is associated with more frequently engaging with professional interpreters for residents [19] and improved skills during clinical simulations for medical students [20]. However, not all institutions provide training, and for those that do, the curricular content can vary widely from simulated patient cases to online videos [19,21]. Although schools are rapidly developing novel telehealth curricula to prepare their trainees for the changing health care landscape, to the authors’ knowledge, no innovations have been published that focus on digital care across language barriers [22-25]. Further, students’ baseline confidence and attitudes around virtual care for patients with LEP, which would help guide the development of such curricula, are unknown.

We set out to examine advanced medical students’ confidence and attitudes toward caring for patients with LEP via telehealth compared with their experiences caring for these patients in person.

## Methods

### Study Design and Participants

This was a cross-sectional survey study of medical students, using a modified version of a survey previously used to assess resident physicians’ experiences working with patients with LEP during in-person interactions [26]. For the medical student survey, we added questions related to telehealth encounters and removed questions that were outside the scope of care provided by medical students. This survey (Multimedia Appendix 1) included questions about respondent characteristics (year in medical school, languages spoken other than English); clinical experience (time spent on clinical rotations at the time of survey, total number of in-person and telehealth encounters with patients with LEP); and any relevant training for caring for patients with LEP outside of the school curriculum. The current curriculum includes a 1-hour lecture on working with interpreters and a 3-hour simulated encounter involving a patient with LEP in which learners communicated with a standardized patient in Spanish or Chinese (Cantonese) by working with nationally certified health care interpreters.

### Survey Administration

This version of the survey was pretested with 2 medical students prior to distribution. The target population of this cross-sectional survey study was third- and fourth-year clerkship medical students at a single US institution. From August to September 2020, we electronically distributed the survey (through Qualtrics) to students who had started clerkships. Our study team sent 4 reminders to encourage participation in the survey, which was voluntary for all students. Students were not required to answer all survey questions.

### Primary Outcomes

The survey included questions on students’ overall confidence in care of patients with LEP. To explore whether students’ confidence would differ when performing clinical tasks of varied complexity, respondents rated their confidence in 8 different clinical tasks for an imagined Amharic-speaking versus English-speaking patient in a telehealth and in-person encounter. These 8 clinical tasks included identifying the patient’s agenda, negotiating visit agenda, assessing medication adherence, developing trust, understanding the patient’s beliefs regarding diabetes mellitus (DM), eliciting patient preferences for DM management, empowering the patient in lifestyle modifications for DM, and incorporating patient preferences and goals in action planning. We asked students to rate their confidence working with a patient with LEP compared with an English-speaking patient using a 5-point Likert scale ranging from very confident to not at all confident. At the time of this study, medical student involvement in telehealth was still in its early stages, making objective assessment challenging; thus, we chose confidence as our outcome based on previous studies in this area and in medical education [26-32].

### Secondary Outcomes

Respondents also compared how frequently they performed 5 different clinical tasks for patients with LEP compared with English-speaking patients during in-person and telehealth encounters. These tasks included performing teach-back, discussing social history details, determining beliefs regarding the management plan, making a personal connection, and asking about nonmedical interests. Students chose from a 5-point Likert scale (ranging from much less often to much more often with LEP patients).

To identify potential explanations for the quantitative findings, in 2 free-response questions, students were asked for their impressions of barriers to caring for patients with LEP in person and in telehealth settings.

### Ethical Considerations

This protocol was approved by the Institutional Review Board of the University of California, San Francisco (#19-29759). All survey responses were completely anonymous. Respondents viewed and agreed with the informed consent statement before proceeding to the first page of survey questions.

### Analysis

All statistical analyses were conducted using SPSS software (version 27; IBM Corp). For all tests, we defined significance as 2-sided *P*<.05.

For our primary outcomes, we used Wilcoxon signed-rank tests to compare students’ overall confidence working with interpreters in telehealth versus in-person settings.

For our secondary outcome of task confidence, we used Wilcoxon signed-rank tests to compare confidence in each of the 8 clinical tasks with patients with and without English proficiency in the telehealth setting, and confidence with care for patients with LEP between in-person and telehealth modalities. For our secondary outcome of relative frequency, we dichotomized the frequency of performing each of the 5 clinical tasks into performing the task less frequently with patients with LEP or equally/more frequently with patients with LEP. We used a chi-square test to compare the relative frequencies of students performing each clinical task during in-person encounters versus telehealth encounters.

We performed bivariate analysis using chi-square tests or Fisher exact tests to identify associations between the outcomes and students’ languages spoken or number of total past encounters with patients with LEP.

To explore potential explanations for our quantitative findings, we summarized the emerging themes from written responses to the free-response questions about barriers to care for patients with LEP in telehealth settings. One author (LY) reviewed all the responses and coded the key barriers using a modified grounded theory methodology [33]; a second author (FN) reviewed the coding. Consensus was reached through discussion and any disagreement was adjudicated by a third author (ECK).

## Results

### Participant Characteristics

A total of 121/300 (40%) medical students responded to the survey. Only respondents who completed at least 50% of survey questions were included in the statistical analysis (n=72; response rate=24%). As not all 72 respondents answered every survey question, we report data using denominators that reflect those who responded to the specific questions analyzed. One respondent had submitted the survey twice. Only data from the survey where this respondent had completed more survey questions were included in the statistical analysis. Among the 72 respondents, 43% (n=31) attended the 3-hour standardized patient encounter approximately 8-9 months prior to completing the survey. Most respondents have had more than 15 in-person encounters with patients with LEP. Conversely, most respondents have had less than 5 telehealth encounters with patients with LEP. Respondent characteristics are provided in Table 1.

**Table 1:** Characteristics of medical student respondents (N=72).

Characteristic				Participants, n (%)
**Year in medical school^a^**				
	Third year			25 (35)
	Fourth year and above			46 (65)
Fluently speaks a non-English language				26 (36)
**Number of encounters with patients with** **limited English proficiency**				
	**In person**			
		≤5 encounters	2 (3)	
		6-15 encounters	19 (27)	
		>15 encounters	51 (71)	
	**Telehealth**			
		≤5 encounters	57 (79)	
		6-15 encounters	14 (20)	
		>15 encounters	1 (1)	

^a^Sample size (N=71; missing=1).

### Primary Outcomes

#### Overall Confidence in Working With Interpreters

Among the 72 respondents, 61% (44/72) were either confident or very confident working with interpreters in person (Table 2). In comparison, respondents were significantly less confident in working with interpreters in telehealth encounters; only 30% (21/72) of respondents were confident or very confident (*P*<.001).

**Table 2:** Respondents’ confidence in working with interpreters in different clinical settings.

		In-person encounters (N=72), n (%)	Telehealth encounters (N=72), n (%)
**Confidence levels**			
	Not at all confident	0 (0)	4 (6)
	Not confident	3 (4)	8 (11)
	Somewhat confident	21 (29)	31 (43)
	Confident	29 (40)	15 (22)
	Very confident	15 (21)	6 (8)

#### Confidence in Performing Patient-Centered Clinical Tasks

**Figure 1:**
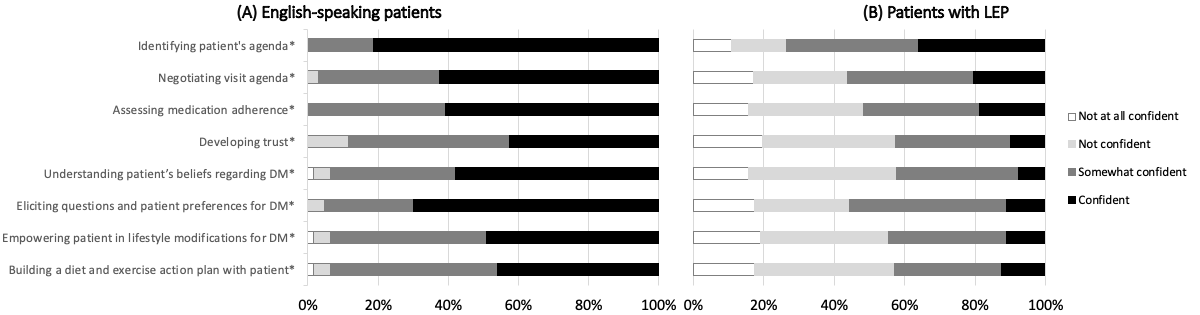
Confidence performing clinical tasks during telehealth encounters. Comparing medical students' self-reported confidence in performing 8 patient-centered tasks in the telehealth setting when working with patients with LEP versus English-speaking patients. Graphs show the percentage of respondents who were "confident" in performing each of the 8 tasks with either patient in the telehealth setting. Percentages reflect only those who rated their confidence in performing clinical tasks with both patients in the telehealth setting (N=61-64). *P<.001 (Wilcoxon signed-rank test, see Table S1 in Multimedia Appendix 2). DM: diabetes; LEP: limited English proficiency.

At least 40% of the 72 respondents reported confidence in performing each of the 8 tasks for an English-speaking patient during a hypothetical telehealth encounter (Figure 1; see Table S1 in Multimedia Appendix 2 for complete data). In the telehealth setting, respondents were significantly less confident when performing each of the 8 clinical tasks with a patient with LEP than with an English-speaking patient (*P*<.001). Less than 20% of students reported confidence performing each of the 8 tasks with a patient with LEP, except identifying the patient’s agenda (23/64, 36%). Respondents felt the least confident in developing trust (6/61, 10%) and understanding the patient’s beliefs regarding DM (5/64, 8%) for the patient with LEP.

#### Patients With LEP in Telehealth Versus In-Person Settings

**Figure 2:**
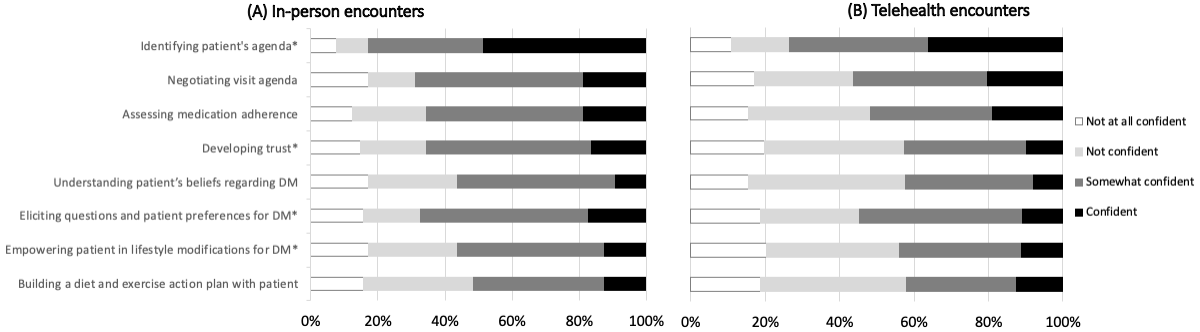
Confidence performing clinical tasks when caring for patients with LEP by clinical setting. Comparing medical students' self-reported confidence in performing 8 patient-centered tasks during in-person versus telehealth encounters with patients with LEP. Percentages reflect only those who rated their confidence in performing clinical tasks in both settings with patients who have LEP (N=61-64). *P<.05 (Wilcoxon signed-rank test, see Table S2 in Multimedia Appendix 2). DM: diabetes; LEP: limited English proficiency.

Across all 8 tasks, a greater proportion of respondents were not confident in working with patients with LEP in a telehealth encounter compared to an in-person encounter (Figure 2). However, these differences were only significant for developing trust (*P*<.001), identifying the patient’s agenda (*P*=.005), eliciting patient preferences for DM management (*P*=.01), and empowering the patient in lifestyle modifications for DM (*P*=.04; see Table S2 in Multimedia Appendix 2 for complete data).

### Secondary Outcomes

#### Frequency of Performing Patient-Centered Clinical Tasks

For both in-person and telehealth encounters, more than 40% of respondents reported completing each of the 5 patient-centered clinical tasks less frequently with patients with LEP than with English-speaking patients (Table 3). Specifically, 78% (46/59) and 66% (39/59) of respondents reported asking about patients’ nonmedical interests less frequently when the patient had LEP during in-person and telehealth encounters, respectively. The distribution of relative frequencies for all 5 tasks did not differ by clinical setting (*P*>.05; see Table S3 in Multimedia Appendix 2 for the chi-square test results).

None of the primary or secondary outcomes discussed above were associated with languages spoken by the respondent or number of previous encounters with patients with LEP (*P*>.05).

**Table 3:** Medical students performing clinical tasks less frequently with patients with LEP compared to English-speaking patients.

Task	In-person encounters, n (%)	Telehealth encounters, n (%)
Perform teach-back	30 (52)	31 (53)
Make a personal connection	26 (44)	27 (46)
Determine beliefs about diagnosis and workup	24 (41)	24 (41)
Discuss details of social history	26 (44)	29 (49)
Asking about patients’ nonmedical interests	46 (78)	39 (66)

#### Barriers to Working With Interpreters: Qualitative Results

A total of 54 of the 72 respondents (75%) answered the qualitative survey questions. All mentioned at least one barrier to in-person care for patients with LEP, including time pressure, interpreter quality and access, technical difficulties, cultural differences, and difficulty with rapport building (Table 4). When asked how these barriers might differ for telehealth encounters, students reported barriers were the same or exacerbated, with specific concerns for the loss of nonverbal cues and physical exam data to inform clinical decision-making as additional barriers. In addition, 24% of respondents (13/54) said they have not had enough telehealth encounters to speak from experience.

**Table 4:** Barriers to care for patients with limited English proficiency.

Themes		Respondent quote
**Time**		
	**Subtheme 1: Additional time needed when working with an interpreter**	
		“Using [working with] an interpreter inherent[ly] prolongs the length of an appointment, oftentimes by more than double what it would take with an English-speaking patient. As such, certain topics that are deemed less essential are often left out in discussions...”
	**Subtheme 2: Direct observation and technical difficulties add to sense of time pressure in telehealth visits**	
		“Sometimes I feel like…on the televisit, the preceptor is watching impatiently (usually I have time in the room alone with the patient and interpreter and don’t feel as rushed).”
**Quality of interpretation**		
		“I speak Spanish and Farsi though I am not certified, so I use [work with] an interpreter each time as required. I have found that occasionally, what I try to communicate is not interpreted as medically desired.”
		“In Spanish which I'm generally accustomed to the visits are quicker, I can understand the patient, I know how the interpreter will interpret…so there is less to wonder about. In other languages it can be harder to know that everyone is on the same page.”
**Cultural differences not mitigated by language**		
		“I was in the room with a provider and a Hindi speaking patient. The patient kept shaking head when provider spoke. In their culture, that means yes…But the provider thought it meant no, disagree and so got frustrated.”
**Access to interpreters: unfamiliar protocols or limited resources**		
		“There are some languages that it is impossible to get an interpreter for in the needed time frame.”
		“We have really struggled to get ASL interpreters for either in-person or telehealth encounters…Some [Deaf patients] have apparently been told to just bring their own interpreter with them.”
**Technical difficulties: with audio, video connection, etc**		
		“Some phone interpreters we cannot hear very well and limit the time for discussion.”
**Building rapport**		
	**Subtheme 1: Difficult when speaking through a third party**	
		“I feel that the personal connection that I am able to build with patients is significantly impaired when I am using [working with] an interpreter despite the fact that I try to follow best practices…”
		“These barriers are similar but magnified [in telehealth] - it's even harder to assess patient understanding and … form a bond/connection with the patient.”
	**Subtheme 2: Deprioritized due to time pressure**	
		“When using [working with] an interpreter the consultation tends to take longer and our encounter, therefore, at times must be more focused and big-picture to make sure we are seeing all clinic patients in a timely manner. There is less time to go through all the details in just one encounter.”
**Navigating own language skills**		
		“…sometimes I have patients say that my Spanish is fine for them… I am just not as fluent as I'd like to be and I worry that patients are too polite to ask for an interpreter after we've already started the visit.”
**Telehealth only: loss of nonverbal cues and objective data to support communication**		
		“With telehealth encounters, you lose body language, eye contact, gestures between you and the patient…and the ability to use physical exam to add to your assessment (if I have less knowledge about their foot injury, I'm less confident communicating it to the patient…).”

## Discussion

### Principal Findings

Our study found that advanced medical students were significantly less confident caring for patients with LEP via telehealth than in-person settings. Moreover, students were significantly less confident developing trust, identifying an agenda, eliciting preferences for management, and empowering patients in lifestyle modifications when caring for patients with LEP virtually compared to in person.

Prior literature has shown that trainees feel less prepared to care for patients with LEP [34]; our findings demonstrate that, although telehealth is a more novel care modality, this gap persists in the virtual setting, and may even be greater, as indicated by the lower confidence reported by participants in this study. Compared with previous studies, however, a greater percentage of students in our sample were confident or very confident in working with interpreters in person [29]. This may reflect institutional differences in education or patient diversity, or a small sample biased toward participation from students interested in culturally and linguistically appropriate care.

A major strength of our study is the breakdown of confidence into specific clinical tasks based on gradation in the complexity of communication skills. Although students reported a lower overall confidence in providing telehealth to patients with LEP, our study provides insights on which specific aspects of the clinical encounter may be more difficult through telehealth. Specifically, tasks such as developing trust or identifying the patient’s agenda and preferences for management may explain the lower confidence while more direct tasks such as assessing medication adherence may be less impacted by the telehealth modality. We found that the overall lower confidence students felt around telehealth care for patients with LEP may be accounted for by some tasks, but not others. According to students’ qualitative responses, loss of nonverbal cues in telehealth is a major barrier; lack of ability to read and portray facial expressions, hand gestures, and other emotional signals may explain perceived challenges with developing trust. This loss is felt more acutely in phone encounters, which patients with LEP are more likely to receive [35]. For the more complex tasks that involve eliciting, processing, and applying information from patients with LEP (eg, eliciting preferences, empowering patients), variation in interpretation quality and time pressure may be barriers to confidence.

### Limitations

This study is limited by the low response rate, small sample size, and single-institution survey, which may restrict a broader application of our findings. Additionally, like many other studies in the field, we have chosen to use a self-reported measure as a proxy for true proficiency [26-29,34]. Although self-assessment is inconsistently correlated with competency [36], there is evidence that providers tend to overestimate their competence working with interpreters [37], suggesting that medical students may be even less prepared to care for patients with LEP than our results have shown. Finally, there may have been factors that we did not account for, such as length of relationship and familiarity with the interpreter, that may influence student confidence.

### Future Directions and Conclusion

In summary, our study demonstrated that self-efficacy and confidence for working with interpreters in the in-person setting were not automatically transferred to the telehealth setting. Additionally, while effective curricula already exist for guiding learners toward best practices for in-person care for patients with LEP, it is unclear whether these curricula are consistently implemented [21]. This lack of education is a possible explanation for why patients with language barriers experience lower quality care [38]. Thus, to better serve our increasingly diversifying patient population, educators should work to adopt these proven curricula while simultaneously building intentional, skills-based sessions [39] that consider the unique challenges that patients with language barriers might face in telehealth encounters. For example, our study highlights several competencies where students may benefit from specific guidance, such as developing patient rapport and cocreating a management plan while working within the constraints of virtual settings. Although this study conducted during the early stages of the COVID-19–driven telehealth expansion used self-competency measures, we recommend that these future interventions be evaluated with knowledge assessments [20], clinical performance scales [40], and other objective tools so we can continue to identify and propagate truly effective curricula.

Finally, it is critical to recognize that provider education is necessary but not sufficient for bridging the gap experienced by linguistically diverse patients [41]. At the policy level, the Joint Commission or other regulatory agencies could develop minimum standards for interpreter quality, including a uniform certification process. Institutions such as universities and hospitals should recruit and support adequate numbers of interpreters as well as bilingual clinicians; this could include appropriate compensation as well as enforcement of universal language access policies across settings. Such steps are essential to ensure that patients with LEP truly receive the best quality of care.

As the COVID-19 pandemic introduces permanent changes to health care delivery, we must ensure that the next generation of providers is prepared to close, not widen, disparities for diverse patient populations.

## Appendix

Multimedia Appendix 1 Survey.

Multimedia Appendix 2 Supplementary tables.

## Abbreviations

DM: diabetes mellitus
LEP: limited English proficiency

## References

[1] Thomas EE, Haydon HM, Mehrotra A, Caffery LJ, Snoswell CL, Banbury A, Smith AC (2022). Building on the momentum: Sustaining telehealth beyond COVID-19. J Telemed Telecare.

[2] Monthly Telehealth Regional Tracker. Fairhealth.

[3] Patel SY, Mehrotra A, Huskamp HA, Uscher-Pines L, Ganguli I, Barnett ML (2021). Trends in outpatient care delivery and telemedicine during the COVID-19 pandemic in the US. JAMA Intern Med.

[4] Katzow MW, Steinway C, Jan S (2020). Telemedicine and health disparities during COVID-19. Pediatrics.

[5] Nouri S, Khoong E, Lyles C, Karliner L (2020). Addressing equity in telemedicine for chronic disease management during the Covid-19 pandemic. NEJM Catal Innov Care Deliv.

[6] Jaffe DH, Lee L, Huynh S, Haskell TP (2020). Health inequalities in the use of telehealth in the United States in the lens of COVID-19. Popul Health Manag.

[7] Selected social characteristics in the United States. US Census Bureau.

[8] Diamond L, Izquierdo K, Canfield D, Matsoukas K, Gany F (2019). A systematic review of the impact of patient-physician non-English language concordance on quality of care and outcomes. J Gen Intern Med.

[9] Lu T, Myerson R (2020). Disparities in health insurance coverage and access to care by English language proficiency in the USA, 2006-2016. J Gen Intern Med.

[10] Ponce NA, Hays RD, Cunningham WE (2006). Linguistic disparities in health care access and health status among older adults. J Gen Intern Med.

[11] Shi L, Lebrun LA, Tsai J (2009). The influence of English proficiency on access to care. Ethn Health.

[12] Rodriguez JA, Saadi A, Schwamm LH, Bates DW, Samal L (2021). Disparities in telehealth use among California patients with limited English proficiency. Health Aff (Millwood).

[13] Eberly LA, Kallan MJ, Julien HM, Haynes N, Khatana SAM, Nathan AS, Snider C, Chokshi NP, Eneanya ND, Takvorian SU, Anastos-Wallen R, Chaiyachati K, Ambrose M, O'Quinn R, Seigerman M, Goldberg LR, Leri D, Choi K, Gitelman Y, Kolansky DM, Cappola TP, Ferrari VA, Hanson CW, Deleener ME, Adusumalli S (2020). Patient characteristics associated with telemedicine access for primary and specialty ambulatory care during the COVID-19 pandemic. JAMA Netw Open.

[14] Eberly LA, Khatana SAM, Nathan AS, Snider C, Julien HM, Deleener ME, Adusumalli S (2020). Telemedicine outpatient cardiovascular care during the COVID-19 pandemic: bridging or opening the digital divide?. Circulation.

[15] Flores G (2005). The impact of medical interpreter services on the quality of health care: a systematic review. Med Care Res Rev.

[16] Karliner LS, Jacobs EA, Chen AH, Mutha S (2007). Do professional interpreters improve clinical care for patients with limited English proficiency? A systematic review of the literature. Health Serv Res.

[17] Parker MM, Fernández A, Moffet HH, Grant RW, Torreblanca A, Karter AJ (2017). Association of patient-physician language concordance and glycemic control for limited-English proficiency Latinos with type 2 diabetes. JAMA Intern Med.

[18] Schulson LB, Anderson TS (2022). National estimates of professional interpreter use in the ambulatory setting. J Gen Intern Med.

[19] Lee KC, Winickoff JP, Kim MK, Campbell EG, Betancourt JR, Park ER, Maina AW, Weissman JS (2006). Resident physicians' use of professional and nonprofessional interpreters: a national survey. JAMA.

[20] Shriner CJ, Hickey DP (2008). Teaching and assessing family medicine clerks' use of medical interpreters. Fam Med.

[21] Himmelstein J, Wright WS, Wiederman MW (2018). U.S. medical school curricula on working with medical interpreters and/or patients with limited English proficiency. Adv Med Educ Pract.

[22] Gordon M, Patricio M, Horne L, Muston A, Alston SR, Pammi M, Thammasitboon S, Park S, Pawlikowska T, Rees EL, Doyle AJ, Daniel M (2020). Developments in medical education in response to the COVID-19 pandemic: A rapid BEME systematic review: BEME Guide No. 63. Med Teach.

[23] Mulcare M, Naik N, Greenwald P, Schullstrom K, Gogia K, Clark S, Kang Y, Sharma R (2020). Advanced communication and examination skills in telemedicine: a structured simulation-based course for medical students. MedEdPORTAL.

[24] Iancu AM, Kemp MT, Alam HB (2020). Unmuting medical students' education: utilizing telemedicine during the COVID-19 pandemic and beyond. J Med Internet Res.

[25] Waseh S, Dicker AP (2019). Telemedicine training in undergraduate medical education: mixed-methods review. JMIR Med Educ.

[26] Williams M, Lewis B, Hauer KE, Fernandez A (2019). Let's work together: residents' perceived ability to communicate management of chronic disease with patients with limited English proficiency. Journal of General Internal Medicine.

[27] Park ER, Chun MBJ, Betancourt JR, Green AR, Weissman JS (2009). Measuring residents' perceived preparedness and skillfulness to deliver cross-cultural care. J Gen Intern Med.

[28] Thompson DA, Hernandez RG, Cowden JD, Sisson SD, Moon M (2013). Caring for patients with limited English proficiency: are residents prepared to use medical interpreters?. Acad Med.

[29] Rodriguez F, Cohen A, Betancourt JR, Green AR (2011). Evaluation of medical student self-rated preparedness to care for limited English proficiency patients. BMC Med Educ.

[30] Shapiro J, Hollingshead J, Morrison E (2003). Self-perceived attitudes and skills of cultural competence: a comparison of family medicine and internal medicine residents. Med Teach.

[31] Gottlieb M, Chan TM, Zaver F, Ellaway R (2022). Confidence-competence alignment and the role of self-confidence in medical education: A conceptual review. Med Educ.

[32] Artino AR (2012). Academic self-efficacy: from educational theory to instructional practice. Perspect Med Educ.

[33] Chapman AL, Hadfield M, Chapman CJ (2015). Qualitative research in healthcare: an introduction to grounded theory using thematic analysis. J R Coll Physicians Edinb.

[34] Weissman JS, Betancourt J, Campbell EG, Park ER, Kim M, Clarridge B, Blumenthal D, Lee KC, Maina AW (2005). Resident physicians' preparedness to provide cross-cultural care. JAMA.

[35] Rodriguez JA, Betancourt JR, Sequist TD, Ganguli I (2021). Differences in the use of telephone and video telemedicine visits during the COVID-19 pandemic. Am J Manag Care.

[36] Davis DA, Mazmanian PE, Fordis M, Van Harrison R, Thorpe KE, Perrier L (2006). Accuracy of physician self-assessment compared with observed measures of competence: a systematic review. JAMA.

[37] Hudelson P, Perneger T, Kolly V, Perron NJ (2012). Self-assessed competency at working with a medical interpreter is not associated with knowledge of good practice. PLoS One.

[38] Green AR, Ngo-Metzger Q, Legedza ATR, Massagli MP, Phillips RS, Iezzoni LI (2005). Interpreter services, language concordance, and health care quality. Experiences of Asian Americans with limited English proficiency. J Gen Intern Med.

[39] Jacobs EA, Diamond LC, Stevak L (2010). The importance of teaching clinicians when and how to work with interpreters. Patient Educ Couns.

[40] Lie D, Bereknyei S, Braddock CH, Encinas J, Ahearn S, Boker JR (2009). Assessing medical students' skills in working with interpreters during patient encounters: a validation study of the Interpreter Scale. Acad Med.

[41] Khoong EC, Fernandez A (2021). Addressing gaps in interpreter use: time for implementation science informed multi-level interventions. J Gen Intern Med.

