# Supplementary material for: Assessing Medical Student Readiness to Navigate Language Barriers in Telehealth: Cross-sectional Survey Study
Source: JMIR Med Educ. 2022 Aug 11;8(3):e36096. doi: 10.2196/36096 (PMC9412902; doi:10.2196/36096)
Supplement: Multimedia Appendix 1 [file mededu_v8i3e36096_app1.pdf]

Default Question Block

UNIVERSITY OF CALIFORNIA, SAN FRANCISCO
CONSENT TO PARTICIPATE IN A RESEARCH STUDY

Impact of preclinical in-person interpreter use training on students' self-efficacy with caring for LEP patients

This is a research study on the effectiveness of a case-based training for medical students on how to work with professional interpreters.

All questions pertain to clinical encounters you have participated in as a medical student during your rotations at UCSF and its affiliated sites.

"LEP patients" refers to patients with limited English proficiency, or patients whose preferred language for communicating health information is not English.

"Telehealth encounter" refers to any encounter where the student/provider and patient were not in the same room, i.e. communicating via phone or video modality.

STUDY SUMMARY

We are asking you to consider taking part in a research study conducted by Leena Yin, Fiona Ng, Dr. Alicia Fernandez, Dr. Elaine Khoong, and Dr. Mia Williams at the University of California, San Francisco.

Purpose of the study: The purpose of this study is to evaluate the impact of a new case-based training for medical students on how to work with professional interpreters.

You are being asked to participate because you are a UCSF medical student who either has or has not experienced this training.

Study Procedures: If you choose to be in this study, you will be prompted to complete a survey and share information about your clerkship encounters with LEP patients. Your participation in the study ends when you complete the survey, which should take about 10 minutes. You can decide to stop at any time by not completing the survey, and your survey responses will not be captured or used for analysis purposes.

Possible Risks: There are minimal risks from taking part in the study as planned.

Possible benefits: You may benefit from participating in the study by taking the time to reflect on your clerkship experiences. Findings from this study may be used to improve preclinical training on how to work with interpreters and care for LEP patients.

Your Other Options: You can choose not to participate without any consequences.

Please access complete consent and description of this study at this link:
https://ucsf.box.com/s/o8dur5r1ny892rtmzhwoz6jnfd9s9077

Please read this description carefully. You can ask the researchers any questions needed to help you decide whether to join the study or not; contact Dr. Elaine Khoong at elaine.khoong@ucsf.edu.

In what year-month did you start clerkships?

Year

Month

How much time have you spent in the hospital or clinic thus far?

< 3 months

At least 3 but less than 6 months

At least 6 but less than 9 months

At least 9 but less than 12 months

12+ months

Among the patients you have cared for during **IN-PERSON** encounters, how many of them have experienced language barriers?

None

1-5

6-10

11-15

Over 15

Please rank the following methods of communicating with LEP patients in the order of how frequently you use each method during **IN-PERSON** encounters (most frequent = 1; least frequent = 6; never used = 0).

	Rank
Get by using my/patient's language skills	
Ask a patient's family member / friend for assistance	
Ask a staff member	
Telephone interpreter	
Video interpreter	
In person interpreter	

Among the patients you have cared for during **TELEHEALTH** encounters**,** how many of them have experienced language barriers?

None

1-5

6-10

11-15

Over 15

Please rank the following methods of communicating with LEP patients in the order of how frequently you use each method during **TELEHEALTH** encounters (most frequent = 1; least frequent = 6;
never used = 0).

	Rank
Get by using my/patient's language skills	
Ask a patient's family member / friend for assistance	
Ask a staff member	
Telephone interpreter	
Video interpreter	
In person interpreter	

The following two questions pertain to identical ***IN PERSON*** clinical situations that differ only in the language and culture of the patient: A 60-year old male patient with type 2 diabetes (DM) and knee osteoarthritis (OA) presents for follow up.

How confident would you feel in carrying out the following tasks? For the Amharic-speaking patient, imagine you have access to the same language resources you would use in your FCM clinic.

(If you speak Amharic, consider the same scenario with a patient who speaks a language that you are not fluent in.)

	English-speaking patient				Amharic-speaking pa		
	Not at all confident	Not confident	Somewhat confident	Confident	Not at all confident	Not confident	Somew confide
Identifying patient's agenda (i.e. knee pain)	O	O	O	O	O	O	O
Negotiating your goals & the patient's goals (i.e. initiating insulin and addressing patient's concern of knee pain)	O	O	O	O	O	O	O

	English-speaking patient				Amharic-speaking pa		
	Not at all confident	Not confident	Somewhat confident	Confident	Not at all confident	Not confident	Somew confide
Assessing patient's adherence to medications	O	O	O	O	O	O	O
Developing trust	O	O	O	O	O	O	O
Understanding patient's beliefs regarding DM	O	O	O	O	O	O	O
Eliciting questions & patient preferences for DM	O	O	O	O	O	O	O
Empowering patient with understanding of importance of lifestyle modifications for DM	O	O	O	O	O	O	O
Building a diet & exercise action plan incorporating patient preferences and goals	O	O	O	O	O	O	O

The following two questions pertain to identical ***TELEHEALTH*** clinical situations that differ only in the language and culture of the patient: A 60-year old male patient with type 2 diabetes (DM) and knee osteoarthritis (OA) presents for follow up.

How confident would you feel in carrying out the following tasks? For the Amharic-speaking patient, imagine you have access to the same language resources you would use in your FCM clinic.

(If you speak Amharic, consider the same scenario with a patient who speaks a language that you are not fluent in.)

	English-speaking patient				Amharic-speaking pa		
	Not at all confident	Not confident	Somewhat confident	Confident	Not at all confident	Not confident	Somew confide
Identifying patient's agenda (i.e. knee pain)	O	O	O	O	O	O	O

	English-speaking patient				Amharic-speaking pa		
	Not at all confident	Not confident	Somewhat confident	Confident	Not at all confident	Not confident	Somew confide
Negotiating your goals and the patient's goals (i.e. initiating insulin and addressing patient's concern of knee pain)	O	O	O	O	O	O	O
Assessing patient's adherence to medications	O	O	O	O	O	O	O
Developing trust	O	O	O	O	O	O	O
Understanding patient's beliefs regarding DM	O	O	O	O	O	O	O
Eliciting questions and patient preferences for DM	O	O	O	O	O	O	O
Empowering patient with understanding of importance of lifestyle modifications for DM	O	O	O	O	O	O	O
Building a diet and exercise action plan incorporating patient preferences and goals	O	O	O	O	O	O	O

For the following questions, compare how often you attempt the following during ***IN PERSON encounters*** with limited English proficiency (LEP) patients in a language in which you are not fluent as compared to with English speaking patients.

	Much less often with LEP patients	Less often with LEP patients	Equal attempts	More with LEP patients	Much more with LEP patients
Ask about patient's non-medical interests	O	O	O	O	O
Discuss details of social history	O	O	O	O	O
Determine beliefs about diagnosis and workup	O	O	O	O	O
Make a personal connection	O	O	O	O	O

	Much less often with LEP patients	Less often with LEP patients	Equal attempts	More with LEP patients	Much more with LEP patients
Perform teach back	○	○	○	○	○

For the following questions, compare how often you attempt the following during ***TELEHEALTH encounters*** with limited English proficiency (LEP) patients in a language in which you are not fluent as compared to with English speaking patients.

	Much less often with LEP patients	Less often with LEP patients	Equal attempts	More with LEP patients	Much more with LEP patients
Ask about patient's non-medical interests	○	○	○	○	○
Discuss details of social history	○	○	○	○	○
Determine beliefs about diagnosis and workup	○	○	○	○	○
Make a personal connection	○	○	○	○	○
Perform teach back	○	○	○	○	○

How satisfied do you feel with the medical care you provide in encounters with limited English proficiency patients in a language in which you are not fluent compared to an English speaking patient?

	Much less satisfied	Less satisfied	Equally satisfied	More satisfied	Much more satisfied
In-Person Encounters	○	○	○	○	○
Telehealth Encounters	○	○	○	○	○

How confident do you feel working with interpreters in clinical encounters?

	Not at all confident	Not confident	Somewhat confident	Confident	Very confident
In-Person Encounters	○	○	○	○	○
Telehealth Encounters	○	○	○	○	○

Briefly, what barriers limit your ability to empower LEP patients with knowledge about their disease, treatment, or medication as compared to English speaking patients during in-person encounters?

Briefly, how are these barriers similar or different in telehealth encounters?

In what non-English language(s) do you consider yourself fluent enough to care for patients without an interpreter? If none, please leave blank.

In the languages above, have you received ALTA certification as a bilingual provider?

Yes, in all the languages listed above

Yes, in some of the languages listed above (if you listed more than one)

No, in none of the languages listed above

I'm not sure / Don't know what this certification is

I did not list any languages above

Did you participate in the CMC Small Group, “Learning to work with limited English proficient patients” in DR block in December 2019?

Yes

No

Don't remember

If you participated in this Small Group, do you have any thoughts or feedback?

Have you received training or have had experience in working with interpreters or LEP patients outside of the Bridges curriculum? *(Note: this can be before or during medical school)*

Case-based group discussion

Practice with standardized patients

Feedback on observed clinical interaction

Role play with peers

Observe video of ideal interaction

Informational reading and/or lecture

No additional training

Other: (please specify)

Create a unique identifier using: First two letters of mother's maiden name, day of your birthday in two digits, first two letters of the city where you lived when you graduated high school.

For example: mother's maiden name (Smith), born on 8th of November (08), and lived in SF when you graduated high school: SM08SF

Powered by Qualtrics
