# Supplementary material for: Assessing Medical Student Readiness to Navigate Language Barriers in Telehealth: Cross-sectional Survey Study
Source: JMIR Med Educ. 2022 Aug 11;8(3):e36096. doi: 10.2196/36096 (PMC9412902; doi:10.2196/36096)
Supplement: Multimedia Appendix 2 [file mededu_v8i3e36096_app2.pdf]

Multimedia Appendix 2

Table S1:

Telehealth confidence level distributions, sample size, and corresponding statistical testing from Figure 1.

	Tasks	Not at all confident N (%)	Not confident N (%)	Somewhat confident N (%)	Confident N (%)	Sample Size n (missing)	Wilcoxon signed rank test (test statistic, z-score, p-value)
1	LEP: Building a diet & exercise action plan with patient	11 (17.5)	25 (39.7)	19 (30.2)	8 (12.7)	n = 63 (9)	
	English: Building a diet & exercise action plan with patient	1 (1.6)	3 (4.8)	30 (47.7)	29 (46.0)	n = 63 (9)	T=0, z=-6.1, p<0.001
2	LEP: Empowering patient in lifestyle modifications for managing diabetes	12 (19.0)	23 (36.5)	21 (33.3)	7 (11.1)	n = 63 (9)	
	English: Empowering patient in lifestyle modifications for managing diabetes	1 (1.6)	3 (4.8)	28 (44.4)	31 (49.2)	n = 63 (9)	T=0, z=-6.1, p<0.001
3	LEP: Eliciting questions & patient preferences for diabetes	11 (17.5)	17 (27.0)	28 (44.4)	7 (11.1)	n = 63 (9)	
	English: Eliciting questions & patient preferences for diabetes	0 (0.0)	3 (4.8)	16 (25.4)	44 (69.8)	n = 63 (9)	T=0, z=-6.3, p<0.001
4	LEP: Understanding patient's beliefs regarding diabetes	10 (15.6)	24 (42.2)	22 (34.4)	5 (7.8)	n = 64 (8)	
	English: Understanding patient's beliefs regarding diabetes	1 (1.6)	3 (4.7)	23 (35.9)	37 (57.8)	n = 64 (8)	T=0, z=-6.3, p<0.001
5	LEP: Developing trust	12 (19.7)	23 (37.7)	20 (32.8)	6 (9.8)	n = 61 (11)	
	English: Developing trust	0 (0.0)	7 (11.5)	28 (45.9)	26 (42.6)	n = 61 (11)	T=0, z=-5.9, p<0.001
6	LEP: Assessing medication adherence	10 (15.6)	21 (32.8)	21 (32.8)	12 (18.8)	n = 64 (8)	
	English: Assessing medication adherence	0 (0.0)	0 (0.0)	25 (39.1)	39 (60.9)	n = 64 (8)	T=0, z=-5.9, p<0.001
7	LEP: Negotiating visit agenda	11 (17.2)	17 (26.6)	23 (35.9)	13 (20.3)	n = 64 (8)	
	English: Negotiating visit agenda	0 (0.0)	2 (3.1)	22 (34.4)	40 (62.5)	n = 64 (8)	T=0, z=-5.7, p<0.001
8	LEP: Identifying patient's agenda	7 (10.9)	10 (15.6)	24 (37.5)	23 (35.9)	n = 64 (8)	
	English: Identifying patient's agenda	0 (0.0)	0 (0.0)	12 (18.8)	52 (81.3)	n = 64 (8)	T=0, z=-5.4, p<0.001

Table S2:

Confidence levels distributions, sample size, and corresponding statistical testing from Figure 2.
Comparing confidence levels when working with patients with LEP during in-person versus telehealth encounters (Figures 1b vs. 2b).

	Task	**Not at all confident** N (%)	**Not confident** N (%)	**Somewhat confident** N (%)	**Confident** N (%)	**Sample Size** n (missing)	**Wilcoxon signed rank test** (test statistic, z-score, p-value)
1	In person: Building a diet & exercise action plan with patient	10 (15.6)	21 (32.8)	25 (39.1)	8 (12.5)	n = 64 (8)	
	Telehealth: Building a diet & exercise action plan with patient	12 (18.8)	25 (39.1)	19 (29.7)	8 (12.5)	n = 64 (8)	T=38.0, z=-1.7, p=0.092
2	In person: Empowering patient in lifestyle modifications for DM	11 (17.2)	17 (26.6)	28 (43.8)	8 (12.5)	n = 64 (8)	
	Telehealth: Empowering patient in lifestyle modifications for DM	13 (20.3)	23 (35.9)	21 (32.8)	7 (10.9)	n = 64 (8)	T=55.0, z=-2.0, p=0.044
3	In person: Eliciting questions & patient preferences for DM	10 (15.6)	11 (17.2)	32 (50.0)	11 (17.2)	n = 64 (8)	
	Telehealth: Eliciting questions & patient preferences for DM	12 (18.8)	17 (26.6)	28 (43.8)	7 (10.9)	n = 64 (8)	T=48.5, z=-2.5, p=0.012
4	In person: Understanding patient's beliefs regarding DM	11 (17.2)	17 (26.6)	30 (46.9)	6 (9.4)	n = 64 (8)	
	Telehealth: Understanding patient's beliefs regarding DM	10 (15.6)	27 (42.2)	22 (34.4)	5 (7.8)	n = 64 (8)	T=36.0, z=-1.8, p=0.077
5	In person: Developing trust	9 (14.8)	12 (19.70	30 (49.2)	10 (16.40	n = 61 (11)	
	Telehealth: Developing trust	12 (19.7)	23 (37.7)	20 (32.8)	6 (9.8)	n = 61 (11)	T=48.0, z=-3.5, p<0.001
6	In person: Assessing medication adherence	8 (12.5)	14 (21.9)	30 (46.8)	12 (18.8)	n = 64 (8)	
	Telehealth: Assessing medication adherence	10 (15.6)	21 (32.8)	21 (32.8)	12 (18.8)	n = 64 (8)	T=71.0, z=-2.0, p=0.051
7	In person: Negotiating visit agenda	11 (17.2)	9 (14.1)	32 (50.0)	12 (18.8)	n = 64 (8)	
	Telehealth: Negotiating visit agenda	11 (17.2)	17 (26.6)	23 (35.9)	13 (20.3)	n = 64 (8)	T=54.0, z=-1.5, p=0.127
8	In person: Identifying patient's agenda	5 (7.8)	6 (9.4)	22 (34.4)	31 (48.4)	n = 64 (8)	
	Telehealth: Identifying patient's agenda	7 (10.9)	10 (15.6)	24 (37.5)	23 (35.9)	n = 64 (8)	T=53.5, z=-2.8, p=0.005

Table S3:

Self-reported relative frequency, sample size, and corresponding statistical testing from Table 3.

	Task	**Less often with LEP patients** N (%)	**Equally or more likely with LEP patients** N (%)	**Sample size n (missing)**	**Chi-squared test** X^2(df) = test statistic, p-value
1	Telehealth: Perform teach back	31 (53.4)	27 (46.6)	n = 58 (14)	
	In person: Perform teach back	30 (51.7)	28 (48.3)	n = 58 (14)	$X^2(1) = 0.03$, p=0.85
2	Telehealth: Make a personal connection	27 (45.8)	32 (54.2)	n = 59 (13)	
	In person: Make a personal connection	26 (44.1)	33 (55.9)	n = 59 (13)	$X^2(1) = 0.03$, p=0.85
3	Telehealth: Determine beliefs about diagnosis and workup	24 (40.7)	35 (59.3)	n = 59 (13)	
	In person: Determine beliefs about diagnosis and workup	24 (40.7)	35 (59.3)	n = 59 (13)	$X^2(1) = 0.0$, p=1.0
4	Telehealth: Discuss details of social history	29 (49.2)	30 (50.8)	n = 59 (13)	
	In person: Discuss details of social history	26 (44.1)	33 (55.9)	n = 59 (13)	$X^2(1) = 0.31$, p=0.58
5	Telehealth: Asking about patients' non-medical interests	39 (66.1)	20 (33.9)	n = 59 (13)	
	In person: Asking about patients' non-medical interests	46 (78.0)	13 (22.0)	n = 59 (13)	$X^2(1) = 2.06$, p=0.15
